# Supplementary material for: Patterns of Intron Gain and Loss in Fungi
Source: PLoS Biol. 2004 Nov 30;2(12):e422. doi: 10.1371/journal.pbio.0020422 (PMC532390; doi:10.1371/journal.pbio.0020422)
Supplement: Table S1 — Also available at http://genes.mit.edu/NielsenEtAl/. (4.3 MB ZIP). [file pbio.0020422.st001.zip › NielsenEtAl/html/412.html]

AN6080.1.NCU02538.1.MG09294.1.FG00505.1


```
 CLUSTAL W (1.82) Multiple Sequence Alignments - Introns Inserted


Sequence 1: NCU02538.1	208 aa
Sequence 2: MG09294.1	203 aa
Sequence 3: FG00505.1	197 aa
Sequence 4: AN6080.1	200 aa
Alignment Length: 209 aa
Number Identitical Residues: 99 aa
Alignment Score (without introns) 5022


MG09294.1 	MAPGMSLSSVNAII~ILGSDDGSRIFAKYYTSPHQAA--~PGAGQAAA1LT~---APPNP
NCU02538.1	MSPGMSLYSVNAIV1ILSSDDGSRIFSKYYTPPHAAAGA~PGTGVAAG~LA1SSSASQNP
FG00505.1 	MSPGMTLFAVQAVL~ILSTEDGSRIFAKYFQPPHSAPNA1PSS-----~--~---ASANP
AN6080.1  	MG-SLSLFSVNAVL~VMSADDGSRIFAKYYSPPHPPAGA~APNSTDY-~--~---PGANP
          	*. .::* :*:*:: ::.::******:**: .** ...: .  .           .  **

MG09294.1 	FPDVKAQKAFEKGLLEKTAKQTGDIILYDNRIVLYKMESDVMMYVVGSVDENEVLLYNVI
NCU02538.1	YPDKTSQTRFEKGLLQKTAKQTGDILLYDNKVVLYKMESDVAIYVVGSADENEVLLYNVL
FG00505.1 	YSDVKAQKAFEKGLIEKTAKQTGDIILYDNRIVLYKMESDVMMYVVGSVDENEILLYNTI
AN6080.1  	YPTLKEQKAFEKGLLEKTNKQTSDVILYDNRIVVFKLESDVMLYVVGGAEENEVLLYNVV
          	:.  . *. *****::** ***.*::****::*::*:**** :****..:***:****.:

MG09294.1 	LALRDSLHLLFK~QSVDKRTIIENYDLVSLAIDEIVDDGIILETDPTIIVQRVSKAPTQD
NCU02538.1	LALRDSLHMLFK2QSIDKRTIVENYDLVSLAIDEIVDDGVVLETDPTIIIQRCSKAPPAD
FG00505.1 	LALRDSLHLLFK2QSVDKRTIVENYDLVSLAIDEIVDDGIILETDPTIIVQRVSRAPTQD
AN6080.1  	LSLRDALGILFK2GATDKRTIVENYDLVALAIDELIDDGIILETDPVLIASRVSRAPQPD
          	*:***:* :***  : *****:******:*****::***::*****.:* .* *:**  *

MG09294.1 	VN-LSRIDPFSEQGVNNLAQLGKAKLTDWLRQGL
NCU02538.1	VN-LSRLDPFTEQGVNNLAQLGKAKLTDWLRQGL
FG00505.1 	MP-VGRID-LSEQGVNNLAQLGKSKLADWLRQGL
AN6080.1  	APNLKSID-LSEQGLLNAWELGKRRLAEGLRQM-
          	  .:  :* ::***: *  :*** :*:: ***
```
